# Is the blood B-cell subset profile diagnostic for Sjögren syndrome?

**DOI:** 10.1136/ard.2008.096172

**Published:** 2008-09-09

**Authors:** A Binard, L Le Pottier, V Devauchelle-Pensec, A Saraux, P Youinou, J-O Pers

**Affiliations:** EA Immunologie et Pathologie, Université Européenne de Bretagne, et Université de Bretagne Occidentale, Brest, et Centre Hospitalier Universitaire de Brest, Brest, France

## Abstract

**Objective:**

To evaluate the relevance of the blood B-cell subset profile for the diagnosis of Sjögren syndrome.

**Methods:**

The distribution of mature blood B cells from Bm1 through Bm5 was determined in 161 patients, of whom 25 fulfilled the American–European Consensus Group criteria for primary SS (pSS), and 136 served as disease controls.

**Results:**

The percentage of Bm2 and Bm2′ cells was increased in the patients with pSS compared with 54 patients with rheumatoid arthritis (RA) and 18 with systemic lupus erythematosus (SLE) (p<0.001 for the two comparisons). In contrast, those of early Bm5 (eBm5) and Bm5 were decreased in patients with pSS, compared with patients with RA and with SLE (p<0.001 for the two comparisons). The receiver operating characteristic curves allowed for an optimising cut-off value of Bm2+Bm2′ cells at 71.1% for 88.0% sensitivity and 83.1% specificity, that of eBm5+Bm5 cells at ⩽13.5% for 84.0% sensitivity and 83.1% specificity, and, consequently, that of Bm2+Bm2′/eBm5+Bm5 at ⩾5 for 88.0% sensitivity and 84.6% specificity.

**Conclusion:**

Given its presentation as a signature for pSS, relative to RA and SLE, such a distribution of B-cell subsets might provide a useful diagnostic tool.

Autoimmune exocrinopathy^1^ is referred to as Sjögren syndrome (SS). This occurs alone as primary SS (pSS), or against a background of connective tissue disease as secondary SS (sSS). Even though keratoconjunctivitis sicca (resulting from the involvement of lachrymal glands) and xerostomia (resulting from the involvement of salivary glands) are usually prominent, SS presents as a multifaceted condition with a broad variety of clinical manifestations and biological abnormalities.

This polymorphism accounts for the delay in the diagnosis, associated with underestimation of the patients’ concerns made by the doctor, which consequently discourages patients from taking medical advice as symptoms arise. As a consequence, there is every likelihood that the prevalence of the disease is far higher than previously estimated.^2^

These disadvantages are being over-ridden by the emergence of new and potentially active treatments, most notably anti-B lymphocyte antibodies (Abs). In the setting of autoimmune diseases, the latter have proved effective in rheumatoid arthritis (RA) and systemic lupus erythematosus (SLE), and are currently being tested in pSS.^3^,^4^ Thus, the need for an early diagnosis of pSS has become a priority. To this end, criteria for classification of the disease have been defined^5^ by the European Community Study Group on diagnostic criteria for SS, and amended^6^ by the American–European Consensus Group (AECG). At this time, special emphasis has been placed on laboratory tests.

Concomitant evidence has sparked a great deal of interest in the possibility that B cells play a leading role in the pathogenesis of pSS.^7^,^8^,^9^,^10^ Among major breakthroughs in this interpretation is the dissection of B-lymphocyte subsets. In particular, the respective membrane expression of IgD and CD38 distributes mature B cells (Bm) into sequential stages from Bm1 through Bm5 cells.^11^ Once activated in secondary lymphoid organs, naïve Bm1 (IgD+/CD38−) become Bm2 (IgD+, CD38+), and progress to germinal centre founder Bm2′ cells (IgD+/CD38++). There, they evolve into Bm3 centroblasts and Bm4 centrocytes (IgD−/CD38+), which differentiate into either plasma cells, or early (eBm5) and late memory Bm5 (IgD−/CD38+ and IgD−/CD38−, respectively). Inside the germinal centres, a few cells of each subset escape into the circulation. For unknown reasons, patients with pSS exhibit disturbed B-cell subset distribution in their blood,^12^,^13^,^14^ and accumulate memory B cells within their exocrine glands.^12^,^13^,^15^

Our aim was to verify whether these disturbances are sufficiently characteristic to provide an additional criterion to those selected^6^ by the AECG. There appeared to be a high ratio of increased percentages of blood Bm2-plus-Bm2′ cells to decreased percentages of eBm5-plus-Bm5 cells that differentiates pSS from RA, SLE and other miscellaneous diseases.

## Patients and methods

### Patients with Sjögren syndrome, disease and normal controls

Details of 177 patients who underwent blood Bm1–Bm5 analysis from January 2002 onwards were reviewed in June 2007. An aliquot was retained for B-cell phenotyping at the time of sampling for serological tests, in connection with routine examination for connective tissue diseases in the rheumatology ward at Brest Medical School Hospital.

A double-blind approach was devised to analyse Bm1–Bm5 distribution. B-cell subsets were studied by the laboratory staff without knowledge of the patients’ diagnosis, which was concurrently established by the clinical staff without knowledge of the Bm1–Bm5 distribution.

Twenty-five patients with an AECG-based diagnosis of pSS,^6^ 54 with an American Rheumatism Association-based diagnosis of RA,^16^ and 18 with an American College of Rheumatology-based diagnosis of SLE were enrolled in the study.^17^ Another 16 patients were classified as undifferentiated arthritis, 11 as osteoarthritis, 8 as fibromyalgia, 7 as spondyloarthropathy, 5 as sicca symptoms of unknown origin, 3 as polymyalgia, 3 as gouty arthritis, 3 as lupus syndrome, and, 1 case each, as viral C hepatitis-associated sicca symptoms, Lyme’s disease, reflex sympathetic dystrophy, low-back pain, lumbar radiculopathy, arthritis-associated paraneoplastic syndrome, unexplained inflammatory syndrome and autoimmune thrombocytopenia. Finally, 16 patients were excluded from the first set of analyses, because SS was secondary (n = 8), lymphoma associated (n = 2), or sufficient clinical details unavailable (n = 6).

Overall, there were three men and 22 women, mean (SD) age 57.2 (13.2) years, with pSS, and 25 men and 111 women, aged 54.2 (14.7) years, with allied autoimmune condition. Among these disease controls, 12 men and 42 women aged 56.0 (12.3) years had RA, and 18 women aged 44.3 (15.0) years had SLE. In addition, 8 male and 18 female healthy volunteers, aged 36.8 (11.6) years, were the normal controls.

### Flow cytometric analysis

Blood mononuclear cells were isolated by Ficoll–Hypaque centrifugation and all Abs were purchased from Beckman-Coulter (Hialeah, Florida, USA). Phycoerythrin-cyanin 7 (PC7)-labelled anti-CD19 monoclonal Ab (J4;119) was used to tag B cells, fluorescein isothiocyanate-labelled IgD (IA6-2) and PC5-labelled CD38 (LS198) to count Bm1–Bm5 subsets.^13^ The cells were categorised on an Epics XL (Beckman-Coulter) fluorescence-activated cell-sorter (FACS).

### Statistical analysis

Quantitative variables were expressed as means (SD). We first used the Mann–Whitney U test to compare the distribution of B-cell subsets in pSS, RA, SLE and NC groups. Then, each case of pSS was classified according to the AECG criteria, and to the B-cell subsets (table 1). The Cohen κ indices were computed to compare AECG-based diagnosis with B-cell subset-based diagnosis. Sensitivities and specificities were calculated for different cut-off values for each B-cell subset levels. Receiver operating characteristic curves were plotted to locate the value nearest the northwest point of these curves which defines the best combination of sensitivity and specificity. When two similar values were obtained the best sensitivity was taken. Calculations were made using the 2007 statistical package for social sciences (SPSS, Chicago, Illinois, USA).

**Table 1:** Clinical features and B-cell subsets of 25 patients with primary Sjögren syndrome (pSS) according the American–European Consensus Group (AECG)

Patients with pSS	AECG items*						Immunosuppressive drugs	Bm2+Bm2′/eBm5+Bm5
	I	II	III	IV	V	VI		
1	+	+	−	+	+	−	MTX 15 mg/week	0.9
							PDN 6 mg/day	
2	+	+	+	−	−	+	−	1.6
3	+	+	−	+	−	+	−	4.3
4	+	+	−	+	−	+	HCQ 400 mg/day	5.0
5	+	+	+	+	+	+	−	5.4
6	+	+	+	+	+	+	−	5.6
7	+	+	+	+	+	−	−	6.0
8	+	+	+	+	−	−	−	6.3
9	+	+	+	+	+	−	HCQ 400 mg/day	7.6
10	+	+	+	+	+	+	HCQ 400 mg/day	7.6
							PDN 5 mg/day	
11	+	−	+	+	+	+	−	7.6
12	+	+	+	+	+	+	−	7.7
13	+	+	+	+	+	−	−	7.8
14	+	+	+	+	+	+	PDN 7 mg/day	8.4
15	+	+	+	−	+	+	−	9.1
16	+	+	+	−	−	+	HCQ 400 mg/day	9.6
17	+	+	−	+	+	+	−	9.6
18	+	+	−	+	−	+	−	12.6
19	+	+	+	−	+	+	−	13.7
20	+	+	−	+	−	+	HCQ 400 mg/day	14.6
21	+	+	+	+	+	+	HCQ 400 mg/day	16.0
22	+	+	+	+	+	+	−	16.3
23	+	+	+	+	+	+	−	18.8
24	−	+	+	+	+	+	−	29.6
25	+	+	+	+	+	+	−	35.2

*AECG items^6^: I, ocular symptoms; II, oral symptoms; III, objective ocular signs (Schirmer’s test ⩽5 mm in 5 min); IV, lip biopsy with focus score ⩾1; V, objective evidence of salivary gland involvement (unstimulated whole salivary flow ⩽1.5 ml in 15 min); VI, anti-SSA and/or anti-SSB. (+), item fulfilled; (−), item not fulfilled.

HCQ, hydroxychloroquine; MTX, methotrexate; PDN, prednisone.

## Results

### B-cell subsets in patients with pSS, RA and SLE, and in normal controls

**Figure 1:**
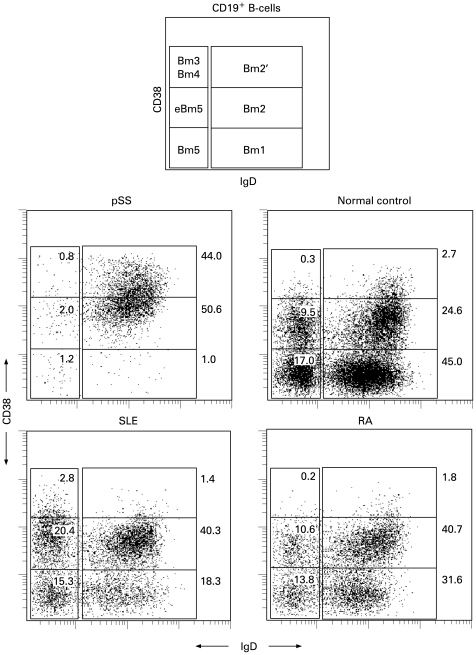
Expression of IgD and CD38 distributes mature B (Bm) cells into sequential subsets from Bm1 through Bm5.^11^ These dot plots are representative examples of fluorescence-activated cell-sorter analysis of patients with primary Sjögren syndrome (pSS), rheumatoid arthritis (RA), or systemic lupus erythematosus (SLE), and of normal controls.

Distribution of blood B-cell subsets was determined in four groups of subjects (table 2); fig 1 shows representative examples. As described,^12^,^13^,^15^ percentages of circulating Bm2+Bm2′ cells were higher in patients with pSS than in normal controls (mean (SD) 77.7 (13.3)% vs 47.3 (4.5)%: p<0.001). Interestingly, they were also higher than for patients with RA (48.8 (17.5)%: p<0.001) and patients with SLE (56.5 (18.5)%: p<0.001). Therefore, the percentages of memory eBm5+Bm5 were decreased in patients with pSS, relative to normal controls (10.9 (7.2)% vs 28.5 (9.1)%: p<0.001), and to patients with RA and SLE (10.9 (7.2)% vs 27.9 (12.0)% and 24.2 (12.7)%, respectively: p<0.001 for both comparisons).

**Table 2:** Blood B-cell subsets or subset combinations (means (SD) of percentage) in patients with primary Sjögren syndrome (pSS), compared with patients with rheumatoid arthritis (RA) or systemic lupus erythematosus (SLE), and with normal controls

B-cell subsets or combinations of subsets	Disease controls			Normal controls(n = 26)
	pSS(n = 25)	RA(n = 54)	SLE(n = 18)	
Bm1*	9.9 (7.5)	20.2 (10.3)	15.2 (14.1)	21.2 (8.6)
		p<0.001	NS	p<0.001
Bm2	65.2 (11.5)	44.8 (15.3)	48.5 (14.7)	43.3 (12.1)
		p<0.001	p<0.001	p<0.001
Bm2′	12.5 (9.9)	4.0 (5.2)	8.0 (7.2)	4.0 (2.7)
		p<0.001	NS	p<0.001
Bm2+Bm2′	77.7 (13.3)	48.8 (17.5)	56.5 (18.5)	47.3 (4.5)
		p<0.001	p<0.001	p<0.001
early Bm5 (eBm5)	5.3 (3.2)	11.0 (4.7)	10.2 (4.9)	13.2 (4.5)
		p<0.001	p<10^−3^	p<0.001
Bm5	5.6 (4.3)	16.9 (9.1)	14.0 (8.9)	15.3 (6.9)
		p<0.001	p<0.001	p<0.001
eBm5+Bm5	10.9 (7.2)	27.9 (12.0)	24.2 (12.7)	28.5 (9.1)
		p<0.001	p<0.001	p<0.001
Bm2+Bm2′/eBm5+Bm5	10.7 (7.9)	2.5 (2.2)	3.4 (2.7)	2.0 (1.1)
		p<0.001	p<0.001	p<0.001

All p values are in comparison with patients with pSS.

*Bm, mature B lymphocyte classified from Bm1 through Bm5, according to the relative membrane expression of IgD and CD38.^11^

### Relevance of B-cell subset profile to the diagnosis of pSS

**Figure 2:**
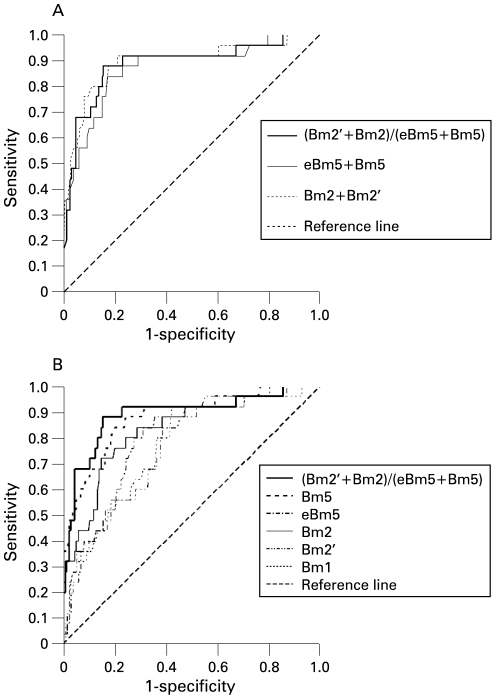
Receiver-operating characteristic curves of B-cell subsets for the diagnosis of primary Sjögren syndrome.

Relationships between AECG-based diagnosis of pSS and B-cell subsets (taken alone or in combination) were evaluated in 25 patients with pSS and 136 disease controls. The best combinations for the diagnosis of pSS (fig 2A, table 3) were Bm2+Bm2′ ⩾71.1% (sensitivity 88.0% and specificity 83.1%), eBm5+Bm5 ⩽13.5% (sensitivity 84.0% and specificity 83.1%), and, even better, the ratio of Bm2+Bm2′ to eBm5+Bm5 ⩾5 (sensitivity 88.0% and specificity 84.6%). Lower associations were seen (fig 2B) with eBm5 ⩽6.7% alone (sensitivity 80.0% and specificity 72.8%), Bm5 ⩽7.6% alone (sensitivity 84.0% and specificity 80.1%), and Bm2 ⩾59.9% alone (sensitivity 80.0% and specificity 75.7%). Parenthetically, the relative percentages of Bm1 and Bm2′ were weakly associated with pSS.

**Table 3:** Optimal levels of B-cell subsets for the diagnosis of primary Sjögren syndrome (in decreasing order)

B-cell subsets	Cut-off point (%)	Sensitivity (%)	Specificity (%)
Bm2+Bm2′/eBm5+Bm5	⩾5.0	88.0	84.6
Bm2+Bm2′	⩾71.1	88.0	83.1
eBm5+Bm5	⩽13.5	84.0	83.1
Bm5	⩽7.6	84.0	80.1
eBm5	⩽6.7	80.0	72.8
Bm2	⩾59.9	80.0	75.7
Bm2′	⩾5.1	84.0	61.8
Bm1	⩽12.9	80.0	64.0

*Bm, mature B lymphocyte classified from Bm1 through Bm5, according to the relative membrane expression of IgD and CD38.^11^

Anti-sicca syndrome (SS)A and anti-SSB Abs were then tested in these 161 subjects. Compared with Bm2+Bm2′/eBm5+Bm5 ⩾5, they had lower sensitivity (76.0% for anti-SSA and 28.0% for anti-SSB vs 88.0% for the ratio of B-cell subsets), but a higher specificity (97.1% for anti-SSA and 97.8% for anti-SSB vs 84.6% for the ratio of B-cell subsets). Association of Bm2+Bm2′/eBm5+Bm5⩾5 with anti-SSA and/or anti-SSB raised the specificity to 99.3%, but reduced the sensitivity to 68.0%.

### Agreement between AECG criteria and Bm2+Bm2′/eBm5+Bm5 ⩾5

AECG-based criteria match the Bm2+Bm2′/eBm5+Bm5 ⩾5 patterns in 137/161 (85.1%) cases (95% confidence interval 78.4 to 90.0 for κ = 0.56). Of the 25 patients fulfilling the AECG criteria for pSS, only three had Bm2+Bm2′/eBm5+Bm5 <5.

Twenty-one cases of Bm2+Bm2′/eBm5+Bm5 >5 were observed in the absence of pSS. These consisted of seven RA, four SLE, three undifferentiated arthritis, two psoriatic arthritis, two fibromyalgia, one sicca syndrome of unknown origin, one gouty arthritis and one osteoarthritis.

### Classification according to B-cell subset, serum and histopathology criteria

**Figure 3:**
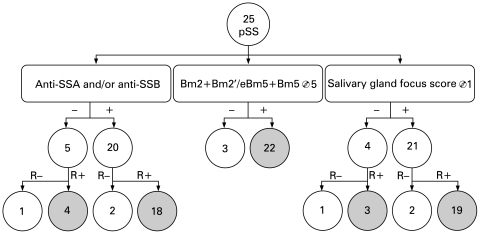
Classification tree for primary Sjögren syndrome (pSS) according to serological, histopathological and phenotypic criteria: the ratio (R) of Bm2+Bm2′ to early Bm5+Bm5 is ⩾5 when denoted “R+”, and <5 when denoted “R−”.

The three criteria, Bm2+Bm2′/eBm5+Bm5 ⩾5, anti-SSA and/or anti-SSB, and focus score ⩾1, were ranked in a classification-tree (fig 3). Of the 25 patients with pSS, 22 had Bm2+Bm2′/eBm5+Bm5 ⩾5, 21 a focus score ⩾1 and 20 anti-SSA and/or anti-SSB.

Within the AECG criteria, the 25 patients displayed anti-SSA and/or anti-SSB and/or a focus score ⩾1. Conversely, among those 22 with Bm2+Bm2′/eBm5+Bm5 ⩾5, 15 had anti-SSA and/or anti-SSB, plus a focus score ⩾1. Three had anti-SSA and/or anti-SSB, but not a focus score ⩾1, while four had a focus score ⩾1, but not anti-SSA and/or anti-SSB. Of the remaining three patients with Bm2+Bm2′/eBm5+Bm5 <5, only one had a focus score ⩾1.

### Circulating B-cell subsets in sSS, or sicca symptoms of unknown origin

Five cases with RA, two with SLE and one with undifferentiated arthritis were clinically classified as sSS. Of these, a first subgroup of four had Bm2+Bm2′/eBm5+Bm5 ⩾5, of whom three fulfilled the AECG criteria for SS. The fourth patient had a focus score ⩾1, but the results of her unstimulated salivary flow were not available. A second subgroup of four patients with sSS had Bm2+Bm2′/eBm5+Bm5 <5, of whom none met the AECG criteria. That is, all the patients who fulfilled the AECG criteria had Bm2+Bm2′/eBm5+Bm5 ⩾5. The B-cell subset distribution patterns might thus be as specific for sSS as for pSS.

By definition, none of five patients with sicca symptoms of unknown origin met the AECG criteria. The anti-SSA and/or anti-SSB Ab tests and the focus score were negative, but one of these five (not the patient with viral C hepatitis-associated sicca symptoms) had Bm2+Bm2′/eBm5+Bm5 ⩾5.

## Discussion

The diagnosis of pSS remains problematic for rheumatologists owing to the lack of specific tests, and therefore its largely subjective approach. This is one of the reasons, not only why the prevalence of the disease varies from one study to another^2^ but also why the proposal of different sets of criteria is never ending.^5^,^6^ Nonetheless, AECG criteria have produced major progresses in this area of research. The latest criteria were indeed based on objective tests, and had forced the experts in the field to reach agreement. Yet, the question is not settled, since doubts can be cast on any item selected.^18^,^19^

Although intended to optimise collaboration between the groups,^20^ these classification criteria are routinely treated as diagnostic criteria. In practice, a patient’s sensitivity and specificity have to be high to allow for an early diagnosis at the beginning of the disease. Furthermore, their improvement is desirable, and the diagnosis worthwhile, given that B-cell depletion could prove a very efficient treatment in the early stages of the disease, but less so in its advanced stages.^3^,^4^

In pSS, there is an increase in the level of Bm2/Bm2′ cells, with a reciprocal decrease in that of eBm5/Bm5. The absence of these two changes in RA and SLE assigns to the blood B-cell subset profile some relevance to the diagnosis of pSS.^13^,^14^ In addition, our analysis establishes that the sensitivity and specificity of just the ratio of Bm2+Bm2′ to eBm5+Bm5 are as good as the entire set of AECG criteria. The sensitivity is higher, and the specificity lower, than anti-SSA and/or anti-SSB. This is remarkable as anti-SSA and/or anti-SSB belong to the AECG criteria set, and, as such, have necessarily been used to validate the inclusion of some patients in this study.

The diagnostic weight of the B-cell subset profile would be even better in a cohort survey than in our cross-sectional analysis. Undoubtedly, the specificity of Bm2+Bm2′/eBm5+Bm5 ⩾5 should be improved in this prospective study, relative to our pilot study. That is, we may safely predict that some cases were false positive, just because the results of a few tests were not available. Actually, some of these patients should have been confirmed as having pSS, and the diagnostic value of Bm2+Bm2′/eBm5+Bm5 improved accordingly. Clearly, this problem warrants examination prospectively in patients with early pSS.

The threat that hangs over patients with pSS is the development of a lymphoma.^21^,^22^ There are currently no reported data relating the alterations in B-cell subsets with the disease duration, inflammatory activity or risk of lymphoma development in pSS. Unfortunately, we have thus far no sufficient data to deal with such pertinent concerns. Consequently, a new prospective study evaluating parotid enlargement and/or palpable purpura and/or reduced levels of C4^22^ for each patient is currently being conducted. This is an attempt to compare patients at “high risk” and patients at “low risk” of lymphoma development relative to their B-cell subset distribution.

B-cell subset disturbances appear to be a signature for SS. Our finding that the Bm2+Bm2′ to eBm5+Bm5 ratio does not reach 5 in SS is sufficient to set this syndrome apart from other rheumatic autoimmune diseases, where a reduced ratio is exceptional. Interestingly, after rituximab treatment, the original abnormalities of B cells (increased Bm2, and decreased memory B cells) are reproduced over time.^23^ Another three arguments supporting a case for this analysis are first, the FACS analysis required to achieve this classification is simple to do, second, the data can be quickly obtained and finally, the results are reproducible. In a way, this simple test could be more readily available to the community doctor than biopsy of the labial minor salivary glands. Should not this be validated as a diagnostic criterion, as an additional asset for the diagnosis of SS in a multicentre prospective study?

## References

[1] Moutsopoulos HM Sjögren’s syndrome: autoimmune epithelitis. Clin Immunol Immunopathol 1994;72:162–5 8050187 10.1006/clin.1994.1123

[2] Binard A Devauchelle V Fautrel B Jousse S Youinou P Saraux A Epidemiology of Sjögren’s syndrome: where are we now? Clin Exp Rheumatol 2007;25:1–4 17417982

[3] Pijpe J van Imhoff GW Spijkervet FK Roodenburg JL Wolbink GJ Mansour K Rituximab treatment in patients with primary Sjögren’s syndrome: an open-label phase II study. Arthritis Rheum 2005;52:2740–50 16142737 10.1002/art.21260

[4] Devauchelle V Pennec Y Morvan J Pers JO Daridon C Jousse S Improvement of Sjögren’s syndrome after two infusions of rituximab. Arthritis Rheum 2007;57:310–7 17330280 10.1002/art.22536

[5] Vitali C Bombardieri S Moutsopoulos HM Balestrieri G Bencivelli W Bernstein RM Preliminary criteria for the classification of Sjögren’s syndrome. Results of a prospective concerted action supported by the European Community. Arthritis Rheum 1993;36:340–7 8452579 10.1002/art.1780360309

[6] Vitali C Bombardieri S Jonsson R Moutsopoulos HM Alexander EL Carsons SE Classification criteria for Sjögren’s syndrome: a revised version of the European criteria proposed by the AECG. Ann Rheum Dis 2002;61:554–8 12006334 10.1136/ard.61.6.554 PMC1754137

[7] Dőrner T Lipsky PE Abnormalities of B cell phenotype, immunoglobulin gene expression and the emergence of autoimmunity in Sjögren’s syndrome. Arthritis Res 2002;4:360–71 12453312 10.1186/ar603 PMC153845

[8] Looney RJ Will targeting B cells be the answer for Sjögren’s syndrome? Arthritis Rheum 2007;56:1371–7 17469093 10.1002/art.22604

[9] Jonsson R Nginamau E Szyszko E Brokstad KA Role of B cells in Sjögren’s syndrome from benign lymphoproliferation to overt malignancy. Front Biosci 2007;12:2159–70 17127453 10.2741/2219

[10] Youinou P Daridon C Steinfeld S Pers JO A case for B cells in the pathogenesis of Sjögren’s syndrome. Current Trends Immunol 2008;8:15–25

[11] Pascual V Liu YJ Magalski A de Bouteiller O Banchereau J Capra JD Analysis of somatic mutation in five B cell subsets of human tonsil. J Exp Med 1994;180:329–39 8006591 10.1084/jem.180.1.329 PMC2191579

[12] Hansen A Odendahl M Reiter K Jacobi AM Feist E Scholze J Diminished peripheral blood memory B cells and accumulation of memory B cells in the salivary glands of patients with Sjögren’s syndrome. Arthritis Rheum 2002;46:2160–71 12209521 10.1002/art.10445

[13] d’Arbonneau F Pers JO Devauchelle V Pennec Y Saraux A Youinou P BAFF-induced changes in BCR-containing lipid rafts in Sjögren’s syndrome. Arthritis Rheum 2006;54:115–26 16385503 10.1002/art.21478

[14] Bohnhorst JO Bjorgan MB Thoen JE Natvig JB Thompson KM Bm1-Bm5 classification of peripheral blood B cells reveals circulating germinal center founder cells in healthy individuals and disturbance in the B-cell subpopulations in patients with primary Sjögren’s syndrome. J Immunol 2001;167:3610–8 11564773 10.4049/jimmunol.167.7.3610

[15] Daridon C Pers JO Devauchelle V Martins-Carvalho C Hutin P Pennec YL Identification of transitional type II B cells in the salivary glands of patients with Sjögren’s syndrome. Arthritis Rheum 2006;54:2280–8 16802367 10.1002/art.21936

[16] Arnett FC Edworthy SM Bloch DA McShane DJ Fries JF Cooper NS The American Rheumatism Association 1987 revised criteria for the classification of rheumatoid arthritis. Arthritis Rheum 1988;31:315–24 3358796 10.1002/art.1780310302

[17] Hochberg MC Updating the ACR revised criteria for the classification of systemic lupus erythematosus. Arthritis Rheum 1997;40:1725 9324032 10.1002/art.1780400928

[18] Manthorpe R Sjögren’s syndrome criteria. Ann Rheum Dis 2002;61:482–4 12006316 10.1136/ard.61.6.482 PMC1754112

[19] Vitali C Classification criteria for Sjögren’s syndrome. Ann Rheum Dis 2003;62:94–5 12480687 10.1136/ard.62.1.94 PMC1754291

[20] Dougados M Gossec L Classification criteria for rheumatic diseases: why and how? Arthritis Rheum 2007;57:1112–5 17907226 10.1002/art.23015

[21] De Vita S Boiocchi M Sorrentino D Carbone A Avellini C Dolcetti R Characterization of prelymphomatous stages of B-cell lymphoproliferation in Sjögren’s syndrome. Arthritis Rheum 1997;40:318–31 9041944 10.1002/art.1780400217

[22] Ioannidis JP Vassiliou VA Moutsopoulos HM Long-term risk of mortality and lymphoproliferative disease and predictive classification of primary Sjögren’s syndrome. Arthritis Rheum 2002;46:741–7 11920410 10.1002/art.10221

[23] Pers JO Devauchelle V Daridon C Bendaoud B Le Berre R Bordron A BAFF-modulated repopulation of B cells in the blood and salivary glands of RTX-treated patients with Sjögren’s syndrome. Arthritis Rheum 2007;56:1464–77 17469105 10.1002/art.22603

